# How Does Subjective Age Get “Under the Skin”? The Association Between Biomarkers and Feeling Older or Younger Than One’s Age: The Health and Retirement Study

**DOI:** 10.1093/geroni/igz035

**Published:** 2019-09-04

**Authors:** Bharat Thyagarajan, Nathan Shippee, Helen Parsons, Sithara Vivek, Eileen Crimmins, Jessica Faul, Tetyana Shippee

**Affiliations:** 1 Department of Laboratory Medicine and Pathology, University of Minnesota, Minneapolis; 2 Division of Health Policy and Management, University of Minnesota School of Public Health, Minneapolis; 3 Davis School of Gerontology, University of Southern California–Davis, Los Angeles; 4 Institute for Social Research, Survey Research Center, University of Michigan, Ann Arbor

**Keywords:** Age discrepancy score, Biological domains, Physiological aging

## Abstract

**Background and Objectives:**

Though subjective age is a well-recognized risk factor for several chronic diseases, the biological basis for these associations remains poorly understood.

**Research Design and Methods:**

We used new comprehensive biomarker data from the 2016 wave of the nationally representative Health and Retirement Study (HRS) to evaluate the association between biomarker levels and self-reported subjective age in a subset of 3,740 HRS participants who provided a blood sample. We measured biomarkers in seven biological domains associated with aging: inflammation, glycemia, lipids, liver function, endocrine function, renal function, and cardiac function. The primary outcome was the age discrepancy score (subjective age − chronological age) categorized as those who felt younger, older, or the same as their chronological age (reference group). Analyses adjusted for comprehensive psychosocial factors (chronic stress index, depression score), demographic factors (race, sex, body mass index, marital status, physical activity), and prevalence of chronic health conditions (comorbidity index).

**Results:**

The prevalence of clinically relevant reduced levels of albumin concentrations was lower in those who felt younger (8.8% vs. 16.0%; *p* = .006) and higher in those who felt older (20.4% vs. 16.0%; *p* = .03) when compared with the reference category. The prevalence of clinically significant elevation in liver enzymes such as alanine aminotransferase was also significantly lower among those who felt younger (7.1% vs. 8.6%; *p* = .04) when compared with the reference category. Prevalence of clinically elevated levels in cystatin C was also lower among those who felt younger when compared with the reference category (50.0% vs. 59.1%; *p* = .04). There was no association between lipids, glucose, or C-reactive protein (inflammatory marker) and subjective age categories.

**Discussion and Implications:**

These results suggest that people who feel younger may have favorable biomarker profiles and as a result may have lower prevalence of age-related diseases when compared with those who feel older or those who feel the same as their chronological age.

**Translational Significance**

Subjective age refers to the difference between how old a person feels compared with their actual age. Biomarkers reflecting nutritional status and liver function are associated with subjective age, suggesting that subjective age can be potentially used as a screening tool for adverse biomarker profiles.

With the unprecedented growth and increasing life expectancy in individuals 65 or older, the U.S. Office of Disease Prevention’s Healthy People plan, HealthyPeople 2020, has prioritized healthy aging; yet, more than 60% of older adults manage two or more chronic conditions (1, 2). Chronological age remains one of the most potent risk factors for a variety of chronic diseases, though there is wide variability in disease prevalence among people of the same chronological age. Hence, investigators have evaluated whether certain psychosocial and biological aspects of aging may moderate the effects of chronological age on various diseases.

Researchers have defined biological age (3) as an alternate summary measure that better reflects overall physiological function when compared with chronological age. A second construct related to aging that has emerged in the psychosocial literature is subjective age, which evaluates an individual’s self-evaluation of how old one perceives oneself to be (4). Though subjective age is a multidimensional construct that includes how old one feels (felt age), how old one would like to be (desired age), and at what age old age begins (perceived age), subjective age is commonly studied as a unidimensional construct with the first construct (felt age) being most extensively studied with regards to health outcomes (5–9). There are two theoretical frameworks to explain why subjective age is associated with a variety of adverse health outcomes including mortality; a social and psychological perspective that views subjective age as being associated with social and environmental cues such as social roles and loneliness (10) and a biomedical perspective that views subjective age as a proxy for individual’s physical health and functioning (11).

In support of the biomedical perspective, prior research has demonstrated that feeling older than one’s chronological age (“older subjective age”) has been found to be associated with several negative health outcomes including increased hospitalization (8), cognitive impairment (5, 6), dementia (6, 7), and higher mortality (9). Furthermore, older subjective age has been associated with increased levels of specific biomarkers such as cystatin C and C-reactive protein (CRP) (12, 13). However, a comprehensive assessment of biological pathways associated with subjective age has not been performed. Hence, it remains unclear whether individuals feel older or younger compared with their chronological age because of their underlying diseases or whether subclinical alterations in biomarkers themselves may be associated with younger or older subjective age. We hypothesize that altered levels of biomarkers that predispose to age-related diseases or biomarkers that change with chronological age will also be associated with older subjective age.

To address this hypothesis, we utilized a novel data source—new comprehensive biomarker data linked with Health and Retirement Study (HRS) data—to assess the relationship between various biomarkers and subjective age. The HRS, a large nationally representative survey, measured large numbers of age-related biomarkers that can be categorized into two broad groups. The first group of biomarkers were those associated with age-related biological process, for example, CRP, which is a commonly used biomarker to estimate systemic inflammation which increases with age (14, 15), dehydroepiandrostenidione (DHEAS), a marker of endocrine function, that has shown to decrease with age (15), or albumin, a marker of nutritional status, that decreases with age (15). The second group of biomarkers were those that are risk factors for age-related diseases, for example, lipid levels that are well-established risk factors for cardiovascular disease (16) or are used to define age-related diseases, fasting glucose is used to identify individuals with diabetes, serum creatinine/cystatin C is used to identify individuals with chronic kidney disease and alterations in liver enzymes are used to diagnose liver dysfunction. The availability of detailed biomarker information along with self-reported prevalence of chronic diseases and a broad range of psychosocial variables in HRS makes this a unique data source to address whether there is an independent association between subjective age and biomarkers after adjustment for psychosocial factors and prevalent chronic diseases. This study will help address whether subjective age can be used as a surrogate for an individual’s physical health and be used to identify individuals at higher risk for age-related chronic diseases and provide further evidence to support the biomedical perspective of subjective age.

## Research Design and Methods

We utilized data from the 2016 HRS survey wave linked to biomarker data measured from venous blood collected from 9,934 participants in 7,227 households during 2016–2017. Among 9,934 participants with biomarkers measured in venous blood, 3,758 participants responded to the question “How old do you feel?” in the 2016 HRS survey that was used to estimate subjective age. After excluding people with missing covariates (*n* = 18), 3,740 participants were included in the final analysis.

Our key independent variables were nineteen biomarkers in seven biological domains that have been previously associated with aging; inflammation (high sensitivity CRP, white blood cell (WBC) count, and neutrophil-to-lymphocyte ratio [NLR]), glycemic indices (fasting glucose), lipids (total cholesterol, high-density cholesterol [HDL-c], low-density cholesterol [LDL-c], and triglycerides), liver function (total protein, albumin, bilirubin, aspartate aminotransferase [AST], alanine aminotransferase [ALT], and alkaline phosphatase), endocrine function (DHEAS, renal function [creatinine, cystatin C, and blood urea nitrogen [BUN]), and cardiac function (N-terminal pro-B-type-natriuretic peptide [NT-proBNP]). A detailed description of these biomarkers has been published previously (17). Chronic ongoing stress index (continuous variable) was a composite score of eight domains of current and ongoing problems lasting 12 months or longer and included (i) ongoing health problems (self), (ii) ongoing emotional or physical problems (spouse or child), (iii) ongoing problems with alcohol or drug use in family member, (iv) ongoing difficulties at work, (v) ongoing financial strain, (vi) ongoing housing problems, (vii) ongoing problems in close relationship, and (viii) helping at least one sick, limited, or frail family member or friend on a regular basis. Each Chronic ongoing stress index domain was rated on a 4-point scale: 1 = “no”; 2 = “yes, but not upsetting”; 3 = “yes, somewhat upsetting”; and 4 = “yes, very upsetting” (18, 19). Physical activity (categorical variable) was a dichotomous variable that was created using a combination of two questions that asked about how often HRS participants played sports/exercised or walked for at least 20 minutes. HRS participants who reported playing sports or exercising or walking at least 20 minutes daily or several times a week were categorized as physically active and others were categorized as physically inactive. CES-D (continuous variable) was a summary score of eight domains such as feeling depressed, felt activities were efforts, sleep was restless in previous week, happy in previous week, felt loneliness in previous week, enjoyed life in previous week, felt sad in previous week, and felt unmotivated in previous week. Participants chose “Yes” (1) to indicate that the depressive symptom had been present during the previous week or “No” (0) to indicate that it had not been present during that time. Two items tapping positive effect (happy in previous week and enjoyed life in previous week) were reverse coded. The CES-D score consisted of a sum of “Yes” responses (20). Comorbidity index was a count of several self-reported chronic diseases such as hypertension, cancer, lung disease, cardiac disorders, stroke, arthritis, and psychiatric problems. Hence, the comorbidity index (continuous variable) indicated the prevalence of the number of self-reported chronic conditions in HRS. Of note, chronic kidney disease and diabetes that were defined using biomarkers evaluated in this study were not included in the comorbidity index. Marital status was analyzed as a categorical variable with six categories: married, never married, annulled, divorced, widowed, and others. Other measures included demographic factors such as race/ethnicity (non-Hispanic White, Black, and Hispanic), sex, and body mass index (BMI; continuous variable).

### Statistical Analysis

Our primary outcome was the discrepancy score between subjective age and chronological age, with positive values indicating feeling older than one’s chronological age and negative values indicating feeling younger than their chronological age. We divided the discrepancy score into three categories: participants feeling younger (those with a negative discrepancy score); participants who felt the same as their chronological age (discrepancy score of 0; reference group); and participants feeling older (those with a positive discrepancy score). To maintain consistency with published literature on this topic, we hereafter refer to the age discrepancy score as subjective age (younger or older) throughout the manuscript (5–10, 12, 13). We initially performed bivariate analysis evaluating the association between the three categories of the subjective age and 19 individual biomarkers, demographic variables, and psychosocial variables. Next, a multinomial logistic regression (PROC SURVEY LOGISTIC) using sampling weights (to account for sample design), strata, and cluster information (to account for participant selection and neighborhood clustering of the sample) was used to evaluate the association between biomarkers and subjective age after adjusting for race, sex, BMI, chronic ongoing stress index, comorbidity index, physical activity, CES-D score, and marital status. We used marital status to adjust for spouses of HRS index participants who lived in the same household. For the primary analysis, the individual biomarkers were dichotomized into clinically relevant “elevated” and “normal” levels using commonly used clinical cutoffs. The cutoff levels were as follows: CRP > 5 mg/L (21); WBC > 11 × 10^9^/L (22), diabetes defined as fasting blood glucose ≥ 126 mg/dL (21) or having history of diabetes or taking oral medication/insulin to control high blood glucose, total cholesterol > 200 mg/dL (16), HDL-c < 60 mg/dL (16), LDL-c > 100 mg/dL (16), triglycerides > 150 mg/dL (16), albumin < 3 g/dL (23), total protein < 6.4 g/dL (21), total bilirubin > 1.0 mg/dL (21), AST > 37 U/L for males and > 31 U/L for females (21), ALT > 40 U/L in males and > 31 U/L in females (21), alkaline phosphatase > 129 U/L in males and >104 U/L in females (21), DHEAS > 13.4 µmol/L in males and > 11 µmol/L in females (21), cystatin C > 1.05 mg/dL (24), BUN > 23 mg/dL (21), grade IV chronic kidney disease = estimated glomerular filtration rate < 15 mL/min based on CKD-Epi equation for serum creatinine (25), NT-Pro-BNP > 125 pg/mL if age < 75 years and > 450 pg/mL if age ≥ 75 years (21). In addition, we also analyzed the individual biomarkers as continuous measures and present the associations for a 1 *SD* increase in biomarker concentration (Table 3). Because there is no clinically accepted reference range for NLR, this biomarker was analyzed only as a continuous variable. Finally, we used the median number of clinically altered biomarkers in the HRS population to dichotomize the frequency of clinically elevated biomarkers into those with 0–5 clinically altered biomarkers and those with 6–12 clinically altered biomarkers. We evaluated the association between the number of clinically altered biomarkers with subjective age using the models described earlier.

## Results

Table 1 shows sample demographic characteristics and biomarker levels by subjective age group. The average discrepancy score between subjective age and chronological age among those who felt younger (*N* = 2,802) was −14.79 years (*SD*: ± 9.22 years) and skewed left with a skewness of −1.59, whereas the discrepancy score for those who felt older (*N* = 402) was 7.46 ± 7.35 years skewed right with a skewness of 1.90. Most people (75%) felt younger than their chronological age, with 14% feeling the same, and another 11% feeling older than their chronological age. Hispanic/Latino adults were more likely to feel older when compared with their Black and non-Hispanic White counterparts. BMI and chronic stress were significantly higher among those who felt older when compared with those who felt younger. HDL-c and serum albumin were lower among those who felt older when compared with those in the reference category and higher in those who felt younger. In contrast, serum triglycerides were higher among those who felt older and lower among those who felt younger when compared with the reference category. The frequency of those who had 6–12 clinically elevated biomarkers was 18.12% among those who felt younger, 30.11% among those in the reference category, and 35.02% among those who felt older (*p* < .0001).

**Table 1.** Distribution of Demographic Characteristics and Biomarker Levels by Subjective Age Group Among 2016 Health and Retirement Survey Respondents With Biomarkers (*N* = 3,740)

		Age Discrepancy Score			
Predictors (Units)	All Participants in Study Cohort	Feeling Younger Than an Individual’s Chronological Age	Feeling the Same as an Individual’s Chronological Age (Reference)	Feeling Older Than an Individual’s Chronological Age	*p* Value
*N*	3,740	2,802 (74.92%)	536 (14.33%)	402 (10.75%)	
Demographics					
Sex (% female)	2,078 (55.57%)	1,590 (56.53%)	272.50 (53.38%)	215.70 (51.77%)	.19
Race					.02
Hispanic	259.50 (6.94%)	194.94 (6.93%)	30.88 (6.05%)	33.67 (8.08%)	
Black	326.30 (8.72%)	225.16 (8.01%)	61.96 (12.14%)	39.18 (9.40%)	
Non-Hispanic White	3,154 (84.34%)	2,393 (85.07%)	417.65 (81.81%)	343.79 (82.51%)	
Smoking status					.004
Never	1,567 (43.30%)	1,230 (45.07%)	197.38 (40.57%)	139.99 (34.63%)	
Former	1,644 (45.42%)	1,208 (44.26%)	237.78 (48.87%)	198.27 (49.05%)	
Current	408.29 (11.28%)	290.91 (10.66%)	51.40 (10.56%)	65.97 (16.32%)	
Physical activity (% active)	2,030 (54.53%)	1,626 (58.07%)	244.18 (48.12%)	159.63 (38.51%)	<.0001
Marital status					.004
Never married	225.09 (6.02%)	146.06 (5.19%)	30.94 (6.06%)	48.09 (11.54%)	
Married	2,265 (60.57%)	1,727 (61.40%)	298.34 (58.44%)	239.95 (57.59%)	
Widowed	614.18 (16.42%)	458.72 (16.31%)	106.86 (20.93%)	48.61 (11.67%)	
Divorced	629.57 (16.83%)	476.78 (16.95%)	74.36 (14.57%)	78.43 (18.82%)	
Other	4 (0.16%)	4.35 (0.15%)	0	1.57 (0.38%)	
Chronological age (y)	68.73 ± 0.33	68.86 ± 0.35	70.22 ± 0.75	66.01 ± 0.64	<.0001
Body mass index (kg/m^2^)	29.42 ± 0.32	28.63 ± 0.27	31.57 ± 1.54	32.12 ± 0.67	<.0001
Chronic Ongoing Stress Score	12.8 ± 0.12	12.41 ± 0.11	12.71 ± 0.30	15.63 ± 0.32	<.0001
CES-D score (depression measure)	1.27 ± 0.05	1.02 ± 0.05	1.50 ± 0.12	2.73 ± 0.18	<.0001
Comorbidity Index	2.01 ± 0.03	1.87 ± 0.03	2.38 ± 0.09	2.55 ± 0.08	<.0001
Markers of inflammation (mean ± *SE*)					
C-reactive protein (high sensitivity) (mg/L)	4.81 ± 0.19	4.43 ± 0.23	5.79 ± 0.63	6.16 ± 0.83	.10
White blood cell count (10^9^/L)	6.72 ± 0.05	6.66 ± 0.05	6.94 ± 0.13	6.81 ± 0.13	.57
Neutrophil-to-lymphocyte ratio	2.29 ± 0.03	2.25 ± 0.03	2.45 ± 0.11	2.37 ± 0.1	.13
Glycemic marker (mean ± *SE*)					
Fasting glucose (mg/dL)	108.91 ± 0.99	107.2 ± 1.04	108.67 ± 1.98	120.83 ± 4.54	.24
Lipid markers (mean ± *SE*)					
Total cholesterol (mg/dL)	189.92 ± 0.81	190.68 ± 0.91	185.74 ± 1.81	189.95 ± 2.82	.53
HDL-cholesterol (mg/dL)	58.42 ± 0.54	59.71 ± 0.59	56.04 ± 1.26	52.63 ± 1.24	.0001
LDL-cholesterol (mg/dL)	102.94 ± 0.69	103.22 ± 0.78	100.84 ± 1.69	103.7 ± 2.26	.92
Triglycerides (mg/dL)	145.82 ± 2.85	141.85 ± 2.32	144.9 ± 3.65	174.15 ± 12.98	.01
Markers of liver function (mean ± *SE*)					
Albumin (g/dL)	3.97 ± 0.01	3.99 ± 0.01	3.93 ± 0.02	3.88 ± 0.03	.01
Protein, total (g/dL)	6.87 ± 0.01	6.88 ± 0.01	6.86 ± 0.02	6.86 ± 0.03	.08
Bilirubin, total (mg/dL)	0.48 ± 0.01	0.48 ± 0.01	0.48 ± 0.01	0.45 ± 0.02	.57
Aspartate aminotransferase (U/L)	22.87 ± 0.33	22.54 ± 0.3	22.68 ± 0.59	25.45 ± 2.02	.30
Alanine aminotransferase (U/L)	21.34 ± 0.37	20.88 ± 0.33	20.84 ± 0.72	25.06 ± 2.14	.12
Alkaline phosphatase (U/L)	81.74 ± 0.82	80.35 ± 0.81	83.52 ± 2.05	89.07 ± 4	.06
Endocrine marker (mean ± *SE*)					
Dehydroepiandrosterone sulfate (µmol/L)	2.28 ± 0.04	2.31 ± 0.05	2.07 ± 0.09	2.30 ± 0.14	.84
Markers of renal function (mean ± *SE*)					
Creatinine (mg/dL)	0.95 ± 0.01	0.93 ± 0.01	1.02 ± 0.03	0.98 ± 0.04	.68
Cystatin C (mg/L)	1.16 ± 0.01	1.13 ± 0.01	1.27 ± 0.03	1.23 ± 0.04	.66
Urea nitrogen (BUN) (mg/dL)	17.97 ± 0.18	17.82 ± 0.16	19.09 ± 0.46	17.63 ± 0.43	.40
Markers of cardiac function (mean ± *SE*)					
N-terminal pro B-type natriuretic peptide (pg/mL)	325.94 ± 24.06	287.95 ± 21.89	546.76 ± 118.92	313.3 ± 40.45	.98

### Association Between Subjective Age and Clinically Relevant Elevations in Biomarkers

In the multinomial logistic regression, the prevalence of diabetes (as defined by a fasting glucose value ≥ 126 mg/dL or having history of diabetes or taking oral medication/insulin to control high blood glucose) was not significantly associated with younger or older subjective age (29.4% vs. 38.4%; *p* = .14; Table 2). Clinically relevant elevations in total cholesterol, HDL-c, LDL-c, and triglycerides were not significantly associated with younger or older subjective age (Table 2). The prevalence of clinically elevated levels in inflammatory biomarkers such as CRP (>5 mg/dL) was also not significantly associated with younger or lower subjective age (Table 2). The prevalence of clinically relevant lower levels of albumin concentrations (<3.5 mg/dL), a marker of nutritional status, was lower in those who felt younger (8.8% vs. 16.0%; *p* = .006) and higher in those who felt older (20.4% vs. 16.0%; *p* = .03) when compared with the reference category. The prevalence of clinically higher levels in liver enzymes such as ALT was also significantly lower among those who felt younger (7.1% vs. 8.6%; *p* = .04) when compared with the reference category. Prevalence of clinically higher levels in cystatin C was also lower among those who felt younger when compared with the reference category (50.0% vs. 59.1%; *p* = .04). Though not statistically significant, the prevalence of clinically significant higher levels in BUN was lower in those who felt older when compared with the reference category (14.18% vs. 18.66%; *p* = .09). Finally, clinically relevant alterations in 6–12 biomarkers had 41% lower odds of feeling younger when compared with the reference category (odds ratio [OR]: 0.64, 95% confidence interval [CI]: 0.47–0.86; *p* < .004), whereas there was no significant association between those who felt older and clinically relevant alterations in biomarkers when compared with the reference group (OR: 1.08, 95% CI: 0.68, 1.71, *p* = .74).

**Table 2.** Prevalence and Association of Clinically Relevant Biomarkers and Age-Related Chronic Diseases across Subjective Age Categories Among 2016 Health and Retirement Survey Respondents With Biomarkers (*N* = 3,740)

Prevalence of Disease Conditions/Clinically Significant Altered Biomarker Concentrations^a^	Feeling Younger Than an Individual’s Chronological Age vs. Reference (*N* = 2,802)			Feeling the Same as an Individual’s Chronological Age (Reference) (*N* = 536)	Feeling Older Than an Individual’s Chronological Age vs. Reference (*N* = 402)		
	Prevalence	OR (95% CI)	*p* Value	Prevalence	Prevalence	OR (95% CI)	*p* Value
	*N* (%)			*N* (%)	*N* (%)		
Markers of inflammation							
C-reactive protein (high sensitivity)	633 (22.59)	0.85 (0.67–1.09)	.20	149 (27.80)	127 (31.59)	0.93 (0.63–1.39)	.73
White blood cell count	80 (2.86)	1.02 (0.38–2.72)	.96	10 (1.87)	17 (4.23)	0.85 (0.25–2.87)	.79
Glycemic marker							
Diabetes (%)	825 (29.44)	0.77 (0.55–1.09)	.14	206 (38.43)	170 (42.29)	0.90 (0.59–1.36)	.60
Lipid markers							
Total cholesterol	1,043 (37.22)	1.08 (0.86–1.37)	.51	174 (32.46)	139 (34.58)	1.01 (0.68–1.49)	.97
HDL-cholesterol	1,646 (58.74)	0.79 (0.59–1.06)	.11	363 (67.72)	280 (69.65)	1.05 (0.71–1.57)	.80
Triglycerides	957 (34.15)	0.90 (0.70–1.16)	.39	203 (37.87)	167 (41.54)	1.00 (0.67–1.50)	.99
LDL-cholesterol	1,328 (47.39)	0.89 (0.70–1.13)	.32	236 (44.03)	183 (45.52)	0.88 (0.58–1.34)	.55
Markers of liver function							
Albumin	246 (8.78)	0.64 (0.47–0.88)	.006	86 (16.04)	82 (20.40)	1.78 (1.06–2.97)	.03
Protein, total	436 (15.56)	0.97 (0.67–1.42)	.88	90 (16.79)	77 (19.15)	1.38 (0.84–2.26)	.20
Bilirubin, total	82 (2.93)	0.87 (0.50–1.53)	.63	24 (4.48)	15 (3.73)	1.48 (0.67–3.29)	.33
Aspartate aminotransferase	163 (5.82)	0.65 (0.36–1.15)	.14	40 (7.46)	35 (8.71)	0.99 (0.49–2.00)	.97
Alanine aminotransferase	200 (7.14)	0.61 (0.38–0.97)	.04	46 (8.58)	44 (10.95)	0.91 (0.49–1.69)	.77
Alkaline phosphatase	286 (10.21)	0.85 (0.50–1.43)	.53	74 (13.81)	54 (13.43)	0.65 (0.31–1.36)	.25
Endocrine marker							
Dehydroepiandrosterone sulfate	4 (0.14)	1.34 (0.10–18.91)	.82	1 (0.19)	1 (0.25)	0.31 (0.01–6.67)	.45
Markers of renal function							
Cystatin C	1,400 (49.96)	0.74 (0.55–0.98)	.04	317 (59.14)	212 (52.74)	0.77 (0.50–1.18)	.22
Blood urea nitrogen	384 (13.70)	0.90 (0.68–1.19)	.44	100 (18.66)	57 (14.18)	0.71 (0.8–1.05)	.09
Chronic kidney disease (%)	10 (0.36)	0.79 (0.14–4.35)	.78	4 (0.75)	7 (1.74)	2.76 (0.44–17.14)	.27
Markers of cardiac function							
NtTerminal pro B-type natriuretic peptide	734 (26.20)	1.11 (0.79–1.56)	.55	171 (31.90)	127 (31.59)	1.11 (0.74–1.67)	.61

*Notes:* CI = confidence interval; OR = odds ratio. ^a^Biomarker cutoff values used to define the clinical threshold. The cutoff levels were as follows: εlevated C-reactive protein = CRP > 5 mg/L. Elevated WBC/immunocompromised = WBC > 11 × 10^9^/L. Prevalence of diabetes = fasting blood glucose ≥ 126 mg/dL or having history of diabetes or taking oral medication/insulin to control high blood glucose. Elevated total cholesterol = total cholesterol > 200 mg/dL. Reduced HDL-cholesterol = HD-cholesterol < 60 mg/dL. Elevated LDL-cholesterol = LDL-cholesterol > 100 mg/dL. Elevated triglycerides = triglycerides > 150 mg/dL. Reduced albumin = albumin < 3 g/dL. Reduced total protein = total protein < 6.4 g/dL (plasma). Elevated total bilirubin = total bilirubin > 1.0 mg/dL. Elevated aspartate aminotransferase > 37 U/L if male and > 31 U/L if female. Elevated alanine aminotransferase > 40 U/L if male and >31 U/L if female. Elevated alkaline phosphatase > 129 U/L if male and >104 U/L if female. Elevated dehydro epiandrosterone sulphate > 13.4 µmol/L if male and >11 µmol/L if female. Elevated cystatin C = cystatin C > 1.05 mg/dL. Elevated BUN = BUN > 23 mg/dL. Grade IV chronic kidney disease = estimated glomerular filtration rate based on CKD-Epi equation for creatinine < 15 mL/min. Elevated B-type N-terminal pro-natriuretic peptide > 125 pg/mL if age <75 y and >450 pg/mL if age ≥ 75 y.

### Association Between Subjective Age and Biomarker as Continuous Variables

Participants with a 1 *SD* higher than the mean fasting glucose levels were also 21% higher odds of feeling older when compared with the reference category (*p* = .01; Table 3). Though there was no significant association between clinically relevant levels of HDL-c, triglycerides, and subjective age, participants with 1 *SD* higher than the mean HDL-c had 17% higher odds of feeling younger (*p* = .05) and 1 *SD* higher than the mean triglycerides had a borderline 16% higher odds of feeling older (*p* = .07; Table 3). Though not statistically significant, a 1 *SD* higher than the mean serum albumin was also associated with 11% greater odds of feeling younger (*p* = .09; Table 3), and this result was consistent with the associations seen between subjective age and clinically relevant elevations in albumin. One *SD* higher than the mean cystatin C was associated with a 10% (*p* = .06) lower odds of feeling younger, whereas 1 *SD* higher than the mean BUN was associated with 14% reduced odds of feeling older (Table 3).

**Table 3.** Association of Clinically Relevant Biomarkers (Continuous Measures) and Age-Related Chronic Diseases across Subjective Age Categories Among 2016 Health and Retirement Survey Respondents With Biomarkers (*N* = 3,740)

Biomarkers	Feeling Younger Than an Individual’s Chronological Age vs. Reference^a^ (*N* = 2,802)	Feeling Older Than an Individual’s Chronological Age vs. Reference^a^ (*N* = 402)
	OR (95% CI); *p* Value	OR (95% CI); *p* Value
Markers of inflammation		
C-reactive protein (high sensitivity)	0.93 (0.84–1.04); .20	1.01 (0.88–1.16); .90
White blood cell count	0.91 (0.80–1.04); .15	0.88 (0.71–1.09);.23
Neutrophil-to- lymphocyte ratio	0.92 (0.78–1.09); .34	0.98 (0.82–1.17);.83
Glycemic marker		
Fasting glucose	1.10 (0.93–1.28); .26	1.21 (1.05–1.40); .01
Lipid markers		
Total cholesterol	1.02 (0.92–1.13); .67	1.02 (0.87–1.20); .82
HDL-cholesterol	1.17 (1.00–1.36); .05	0.91 (0.69–1.19); .49
LDL-cholesterol	0.97 (0.86–1.09); .59	0.97 (0.81–1.16); .74
Triglycerides	1.01 (0.91–1.12); .90	1.16 (0.99–1.36); .07
Markers of liver function		
Albumin	1.11 (0.98–1.26); .09	0.88 (0.74–1.05); .15
Protein, total	1.03 (0.91–1.18); .62	0.88 (0.72–1.08); .21
Bilirubin, total	1.05 (0.91–1.20); .49	0.89 (0.73–1.10); .27
Aspartate aminotransferase	0.95 (0.84–1.07); .37	1.06 (0.91–1.23); .44
Alanine aminotransferase	0.94 (0.83–1.06); .31	1.09 (0.94–1.26); .24
Alkaline phosphatase	0.93 (0.79–1.10); .38	1.01 (0.83–1.22); .94
Endocrine marker		
Dehydroepiandrosterone sulfate	1.10 (0.93–1.30); .27	1.02 (0.81–1.27); .89
Markers of renal function		
Creatinine	0.95 (0.86–1.06); .36	0.98 (0.85–1.14); .82
Cystatin C	0.90 (0.81–1.00); .06	0.97 (0.83–1.13); .67
Urea nitrogen (BUN)	0.93 (0.84–1.03); .13	0.86 (0.73–1.01); .07
Markers of cardiac function		
N-terminal pro B-type natriuretic	0.99 (0.89–1.09); .80	0.96 (0.81–1.14); .63

*Notes:* CI = confidence interval; OR = odds ratio. ^a^Reference: Feeling same as an individual’s chronological age.

## Discussion and Implications

This is the first study to use a large, nationally representative survey of older adults with comprehensive biomarker data to evaluate the relationship between biomarkers in seven biological domains and subjective age, a topic of high policy interest (26), while controlling for other relevant factors. We found that poor nutritional status (as estimated by low serum albumin) and clinically elevated levels of renal biomarkers (eg, cystatin C) to be lower in those who felt younger.

These results suggest that markers of nutritional status (eg, albumin) and renal biomarkers (eg, cystatin C) are important factors associated with the discrepancy between subjective and chronological age. This is consistent with published literature where older subjective age is associated with higher levels of cystatin C (13). Though CRP was not associated with subjective age in this study, the direction of effect of similar to that observed in a previous study that showed an association between CRP and subjective age (12). However, this is the first study to show that higher levels of albumin, an analyte associated with adequate nutritional status is positively associated with younger subjective age, whereas elevated levels of ALT, a liver enzyme, is negatively associated with younger subjective age. Because albumin, the most abundant protein in the plasma, is produced exclusively in the liver (27) and higher ALT is indicative of injury to hepatocytes, both these biomarkers may indicate that impaired liver function may have some subtle physical manifestations that may be reflected in an older subjective age. In addition, low albumin levels are also a sensitive indicator of nutritional status and have been shown to be associated with greater disabilities in activities of daily living among community-dwelling adults (27, 28). Thus, albumin levels may reflect poor physical health and an older subjective age may be a proxy for poor physical health. Although cystatin C is most commonly used clinically as a marker of renal function, cystatin C has numerous physiological roles in multiple organs (29). Higher cystatin C levels have been associated with accelerated cognitive decline (30) and cerebral white matter hyperintensities, which are markers of the brain aging process (31). Thus, the higher prevalence of normal levels of cystatin C may be reflective of better cognitive function in those with younger subjective age.

These findings provide support for the biomedical perspective of subjective age in that the discrepancy between subjective age and chronological age may reflect underlying physical and biological function and that younger subjective age may be associated with favorable biomarker profiles that may reflect adequate functioning of various biological pathways (11). In addition, the low frequency of clinically relevant elevated biomarkers among those who felt younger suggests that a younger subjective age is associated with an optimal biomarker profiles in several domains, whereas older subjective age is associated with global dysfunction of multiple biological pathways. Furthermore, these biomarkers were associated with the discrepancy score after adjustment for chronic ongoing stress, which is a summary measure of multiple psychosocial stressors that include stress due to health problems, financial stress, substance abuse, and housing problems. Thus, the observed associations appear to be independent of psychosocial factors that may be associated with subjective age providing further support for a biomedical perspective as to why individuals feel younger or older when compared with their chronological age. Consistent with the observation that most people report feeling younger, we observe the significant differences in biomarker profiles predominantly among those who feel younger when compared with those who feel the same as their chronological age, while there are relatively few significant differences between those who feel older and those who feel the same as their chronological age. The substantially lower prevalence of clinically relevant alterations in biomarker levels suggests that those who feel younger may indeed have favorable biomarker profiles when compared with the other two groups. The finding that feeling older is less common is consistent with existing literature, which show that people generally feel younger than their chronological age (9). This means that feeling the same or older may be associated with physiological deficiencies or other challenges. Statistically, the “feeling older” group is relatively smaller (ie, there was a lower probability of feeling older), which might suggest concerns for testing the associations of other exposures. However, multinomial logistic regression estimates are for the whole sample and are thus superior to separate binary tests of outcome versus reference categories, typically resulting in smaller standard errors than for separate binary tests. Moreover, even our smallest outcome group has *n* = 402, which is well more than 10 cases per covariate. This suggests that, although some group size concerns remain, overall group size imbalance or small group size among the “feeling older” group may be relatively minor explanations for any nonsignificant associations found.

Thus, older subjective age may be a surrogate marker for adverse biomarker profiles, poorer physical health, and a lifestyle that predisposes to chronic diseases. Though subjective age has been shown to be a modifiable concept, at least in the short term (32), modifying the discrepancy between subjective age and chronological age itself may not be a desirable target for intervention. Instead, individuals with an older subjective age are more likely to exhibit clinically significant levels of biomarkers that would identify those at higher risk for age-related chronic diseases. In this regard, asking people a single question “how old they feel?” may serve as an initial indicator to identify people at increased risk for several age-related diseases such as diabetes and chronic kidney disease. In addition to screening for medical diseases, subjective age may serve as an indicator for individuals who may have difficulty in meeting their nutritional needs (as evidenced by association with low albumin) and may provide opportunities to identify people who may benefit from nutritional supplementation.

Limitations of these analyses include the cross-sectional study design that precludes determination of temporality of observed associations. Thus, it is not clear whether differences in biomarker profiles influence subjective age or whether feeling older indirectly influences lifestyle factors and physical health that is reflected in adverse biomarker profiles. So, although these findings do provide some support for the association between biomarkers and subjective age, longitudinal studies where both subjective age, biomarkers, and psychosocial factors measured over time are needed to fully understand the psychosocial and biomedical determinants of subjective age. Furthermore, evaluation of subjective age at a single time point does not provide an estimate of the stability of subjective age over time. Though factors such as depressive mood that influence day-to-day variability in subjective age (33), several studies have shown that the discrepancy between subjective age and chronological age remains relatively stable over 4–8 years in those over the age of 50 years (34–37). Finally, though the chronic stress score is a valid measure of psychological stress, there may be specific domains of psychosocial stress (eg, depressive mood) that is not adequately captured using the summary score and may attenuate the observed association between individual biomarkers and subjective age. However, we included the CES-D score as an independent variable to better adjust for depression in this cohort. Because HRS included couples from the same household, it may be possible that the data obtained from people living in the same household were not independent and may affect the observed associations. We conducted sensitivity analysis, evaluating these associations only among primary respondents (excluding spouses) and found results very similar to those presented in this manuscript (data not shown). Hence, inclusion of individuals from the same household did not influence the results of this study. The entire range of biomarker data are only available for HRS participants who participated in the 2016 wave, precluding any longitudinal analysis of biomarkers and subjective age in HRS. Additional follow-up of these individuals in future HRS surveys will help clarify the temporal association between biomarkers and subjective age. Finally, other physical measures such as grip strength and vision impairments may also contribute to the discrepancy between subjective age and chronological age that were not assessed in this study. Despite these limitations, this is the first study to demonstrate that specific biological domains may be related to subjective age, an easy to measure indicator of psychological and physiological aging that may inform future strategies to reduce morbidity from chronic diseases and mortality among the older adults.
